# Probing primordial gravitational waves: Ali CMB Polarization Telescope

**DOI:** 10.1093/nsr/nwy019

**Published:** 2018-02-05

**Authors:** Hong Li, Si-Yu Li, Yang Liu, Yong-Ping Li, Yifu Cai, Mingzhe Li, Gong-Bo Zhao, Cong-Zhan Liu, Zheng-Wei Li, He Xu, Di Wu, Yong-Jie Zhang, Zu-Hui Fan, Yong-Qiang Yao, Chao-Lin Kuo, Fang-Jun Lu, Xinmin Zhang

**Affiliations:** 1 Key Laboratory of Particle Astrophysics, Institute of High Energy Physics, Chinese Academy of Sciences, Beijing 100049, China; 2 Theoretical Physics Division, Institute of High Energy Physics, Chinese Academy of Sciences, Beijing 100049, China; 3 University of Chinese Academy of Sciences, Beijing 100049, China; 4 CAS Key Laboratory for Researches in Galaxies and Cosmology, Department of Astronomy, University of Science and Technology of China, Hefei 230026, China; 5 Interdisciplinary Center for Theoretical Study, University of Science and Technology of China, Hefei 230026, China; 6 National Astronomical Observatories, Chinese Academy of Sciences, Beijing 100012, China; 7 Institute of Cosmology & Gravitation, University of Portsmouth, Portsmouth PO1 3FX, UK; 8 Department of Astronomy, School of Physics, Peking University, Beijing 100871, China; 9 Physics Department, Stanford University, Stanford, CA 94305, USA

**Keywords:** primordial gravitational waves, cosmic microwave background, Ali CMB Polarization Telescope

## Abstract

In this paper, we will give a general introduction to the Ali CMB Polarization Telescope (AliCPT) project, which is a Sino–US joint project led by the Institute of High Energy Physics and involves many different institutes in China. It is the first ground-based Cosmic Microwave Background (CMB) polarization experiment in China and an integral part of China's Gravitational-wave Program. The main scientific goal of the AliCPT project is to probe the primordial gravitational waves (PGWs) originating from the very early Universe. The AliCPT project includes two stages. The first stage, referred to as AliCPT-1, is to build a telescope in the Ali region of Tibet at an altitude of 5250 meters. Once completed, it will be the highest ground-based CMB observatory in the world and will open a new window for probing PGWs in the northern hemisphere. The AliCPT-1 telescope is designed to have about 7000 transition-edge sensor detectors at 95 GHz and 150 GHz. The second stage is to have a more sensitive telescope (AliCPT-2) with more than 20 000 detectors. Our simulations show that AliCPT will improve the current constraint on the tensor-to-scalar ratio *r* by one order of magnitude with three years' observation. Besides the PGWs, AliCPT will also enable a precise measurement of the CMB rotation angle and provide a precise test of the CPT symmetry. We show that three years' observation will improve the current limit by two orders of magnitude.

## INTRODUCTION

Searching for gravitational waves (GWs) has long been the cornerstone of cosmology and astrophysics since Einstein proposed general relativity (GR) in the early 20th century. GWs are thought to be the last piece of the theoretical predictions of GR. With long-lasting efforts, the LIGO (Laser Interferometer Gravitational-Wave Observatory) Collaboration in 2016 announced the first detection of GWs with signals coming from two merging black holes with a mass of tens of solar masses [1]. Since then, LIGO and Virgo have announced three other events of black hole [2–4] and one event of neutron star GWs [5]. These achievements will allow the study of GWs to enter a new era and were awarded the Nobel Prize in Physics in 2017.

Unlike the GWs detected by LIGO and Virgo, primordial gravitational waves (PGWs) arise from quantum fluctuations and carry important information about the very early Universe, for example, the physics of inflation, bouncing and the emergent Universe. So far the most effective way to probe PGWs is to measure the B-mode polarization of the cosmic microwave background (CMB).

CMB photons are relics left after the Big Bang. Their first detection half a century ago pioneered the study of cosmology. In the recent 20 to 30 years, CMB observations have developed rapidly, leading us into the precision cosmology era. However, the CMB B-mode polarization induced by tensor fluctuations generated in the early Universe, i.e. PGWs, has still not been detected conclusively (in 2014, the BICEP2 Collaboration [6] announced the detection of the B-mode, which, however, turned out to be dominated by dust emissions, not the signal of PGWs [7]). This has become a key scientific goal of CMB observations in recent years. In addition, CMB B-modes provide us with an important test of fundamental physics, such as CPT symmetry.

At present, the major ground-based CMB experiments are in the southern hemisphere, for example, the Atacama Cosmology Telescope (ACT) and the POLARBEAR/Simons Array in Chile, and the South Pole Telescope (SPT) and BICEP at the South Pole. High-precision experiments in the northern hemisphere are critically needed to achieve full sky coverage.

In 2014, the cosmology team at the Institute of High Energy Physics proposed a CMB experiment in Ali in Tibet, aiming to search for PGWs in the northern hemisphere. In this paper we provide a general introduction to the Ali CMB Polarization Telescope (AliCPT) project. In the section entitled ‘Overview of the AliCPT site', we introduce the atmospheric conditions, sky coverage and infrastructure of AliCPT. In the section entitled ‘AliCPT and its scientific goals', we present the main scientific goals of AliCPT. Our summary is presented in the final section.

## OVERVIEW OF THE ALICPT SITE

In this section, we describe the atmospheric conditions, sky coverage and infrastructure of the AliCPT site.

**Figure 1.**
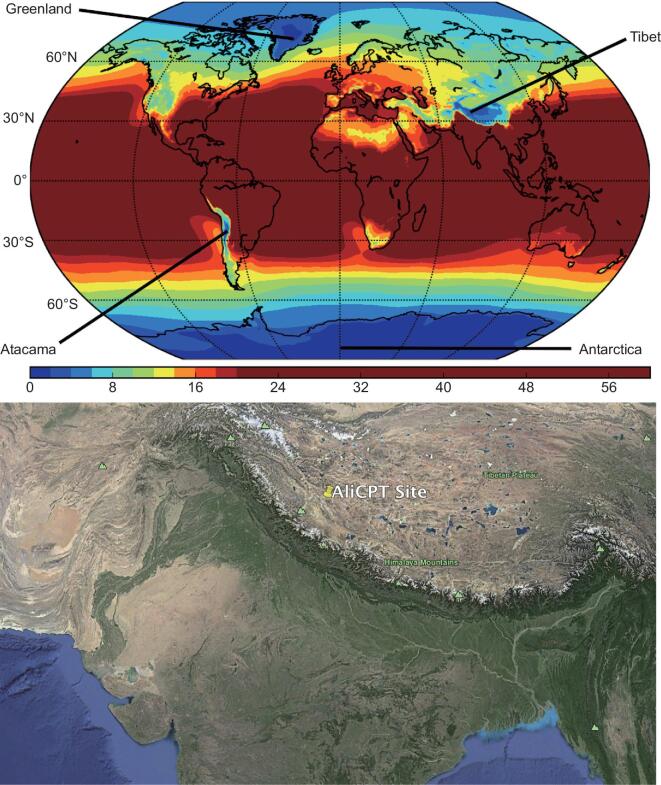
Global distribution of mean PWV over six years (July 2011–July 2017) obtained with MERRA-2 data. The color bar is in millimeters (upper). The location of the AliCPT site and the surrounding terrain. The wet air from the Indian Ocean is hugely reduced by the Himalayan mountains (lower).

**Figure 2.**
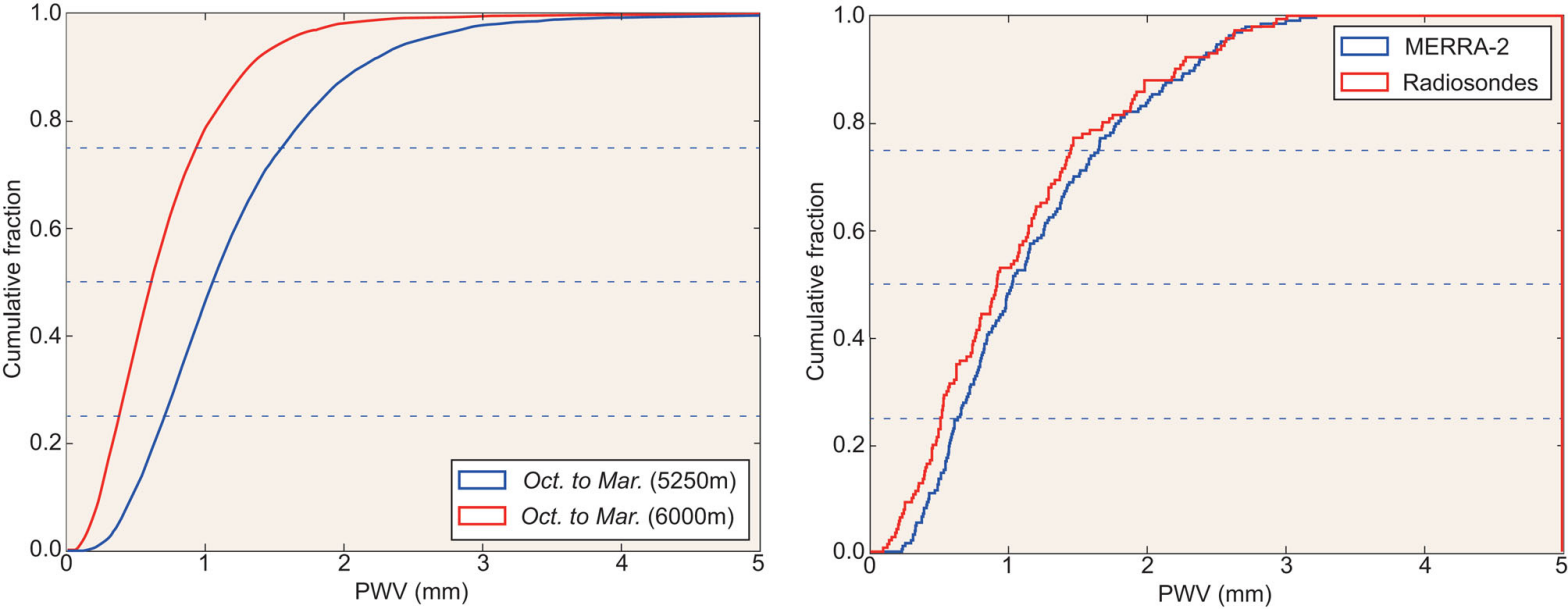
The cumulative distribution of PWV for sites at 5250 m and 6000 m over the observing season obtained with MERRA-2 data (left). The comparison between MERRA-2 and radiosonde datasets at 5250 m (right). Results are taken from [8].

**Figure 3.**
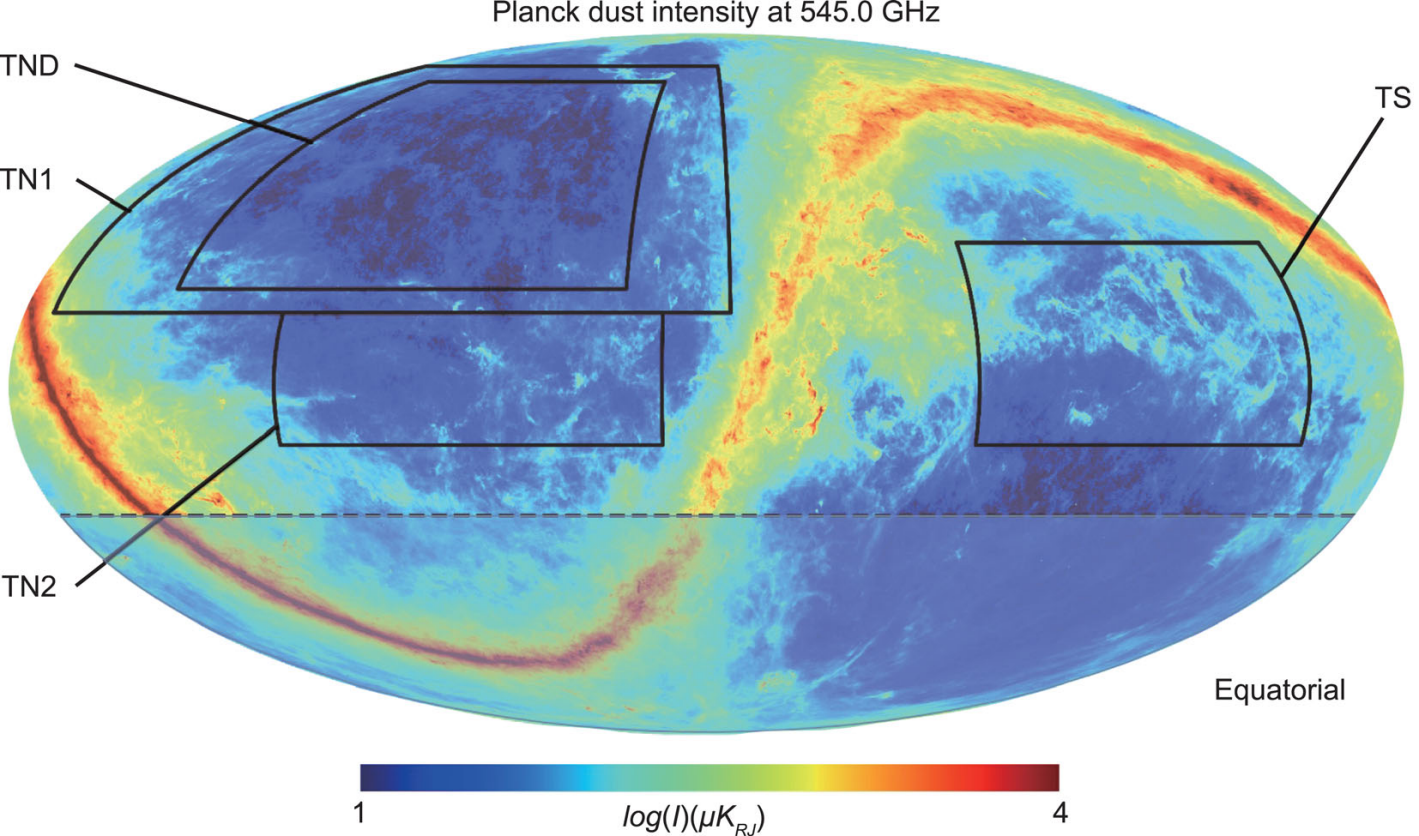
Sky coverage of AliCPT. The observable sky is above the black dashed line; the target fields are shown as TN1 and TN2 in the northern galactic hemisphere and TS in the southern galactic hemisphere. TND is for the deeper survey.

**Figure 4.**
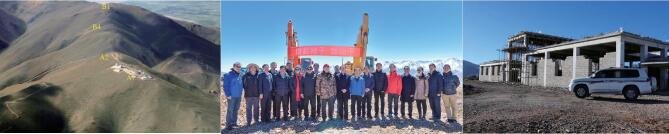
From left to right: aerial view of the region around Ali Astronomical Observatory (5100 m at A1); laying the foundation for AliCPT at point B1 (5250 m); the current infrastructure for AliCPT.

1. **Atmospheric conditions:** For ground-based CMB telescopes, the atmosphere is a big concern. Absorption and emission in the millimeter/sub-millimeter bands by air molecules reduce the significance of signals. Among all the components of air, water vapor plays a crucial role due to its strong absorption/emission and heavy time variation. Usually, the precipitable water vapor (PWV) is used as a conventional parameter to characterize the amount of water vapor, which is defined as the overall depth of water in a column of the atmosphere above the ground. CMB signals, especially polarization signals, are extremely weak, so observation requires the air of the site to be thin, dry and stable. In the upper panel of Fig. 1, we show the global distribution of the mean values of PWV over the past six years (July 2011–July 2017). It is obvious that only four regions have the lowest PWV on earth, including the Antarctic, Atacama Desert, Greenland and the high Tibetan Plateau.
  The current AliCPT site is located on a 5250 m high peak of the Gangdise mountain. Another site nearby with an altitude of about 6000 m has also been considered for the forthcoming project. The Himalayas are to its southwest and run northwest to southeast, separating the Ali area from the Indian subcontinent as well as the Indian Ocean, which is shown in the lower panel of Fig. 1. So the wet air from the Indian Ocean is largely reduced. These factors make the air of the AliCPT site thin and dry enough around winter. In [8], we quantitatively analyzed the atmospheric conditions of the site using radiosonde data from the local weather station as well as MERRA-2 reanalysis data from NASA/GMAO. The results show that the PWV of Ali has a very strong seasonal variation and the median PWV in the observing season (October to March) is about 1 mm (1.07 mm for MERRA-2, 0.92 mm for radiosondes), which is excellent for observation at 95/150 GHz. In Fig. 2 we plot, as a function of PWV, the fraction of time during which the water vapor condition is better than a specific PWV value. Ye *et al.* [9] and Kuo [10] have also evaluated the atmospheric conditions of the Ali region.
2. **Sky coverage:** The AliCPT site is located at the geographical coordinates (80° 01′ 50′ E, 32° 18′ 38″ N). With the rotation of the earth and the mid-latitude location, AliCPT is able to cover the whole northern sky as well as the low-latitude part of the southern sky, and the overall observable fraction is about 70%. We show this in Fig. 3 as the region above the black dashed line. In our calculation we choose the instrumental parameters to be a 45° lowest elevation for the mount and a 30° field of view (FOV). The overlap in the low-latitude region of observable sky of Ali and Atacama makes it convenient to do cross-check and cross-correlation studies. Known as the northern hole, the lowest foreground contaminated region in the northern galactic hemisphere is also within the observable sky of AliCPT, which is extremely important for a CMB B-mode polarization and PGW-aimed project. We show the target fields of AliCPT in Fig. 3. TN1 and TN2 within the black solid lines are target fields in the northern galactic hemisphere; TS is that in the southern galactic hemisphere. TND, with the lowest dust intensity, is chosen for the deeper survey.
  So the sky coverage (both observable and low foreground contamination sky) of AliCPT is complementary to that of the experiments in Antarctic and Atacama. Together with the southern cleanest sky region covered by southern projects such as BICEP and the Simons Array, AliCPT will increase the chances of finding the B-mode and PGWs. For more details about sky coverage, refer to [8].
3. **Infrastructure:** In terms of transportation, National Highway 219 is right next to the AliCPT site. Moreover, Ngari Gunsa Airport is only about half an hour's driving distance from the site and has a daily commercial flight to Lhasa, the capital of Tibet. The largest settlement in the Ali area, the town of Shiquanhe, is also very close to the site, only a 30 minutes' drive away.
  From the aerial view around Ali Astronomical Observatory at point A1 (left panel of Fig. 4), we can see that the AliCPT site at B1 is not far from point A1: the distance between them is only about 1 km. The concrete road from A1 to B1 is already under construction and will be finished soon. In Ali Astronomical Observatory at point A1, city grid electric power and the network infrastructure for data transmission are ready now. Some optical telescopes for astrophysics have been set up. Having started in March 2017, the site construction of AliCPT is ongoing and the main building is now finished. After commissioning in 2019, the observation is expected to start in 2020.

In short, AliCPT opens a new window in the northern hemisphere to detect CMB B-mode polarization and probe PGWs.

## ALICPT AND ITS SCIENTIFIC GOALS

In this section, we provide a brief introduction to the main contents and scientific goals of the AliCPT project. The project consists of two stages. The first stage is to develop and deploy a CMB polarization telescope at 5250 meters, called AliCPT-1. The AliCPT-1 telescope is a dichroic refractor of aperture 70 cm covering 95/150 GHz, with a three-axis driving mount scanning at a speed of 5°/s in azimuth. AliCPT-1 adopts transition-edge sensor (TES) bolometers used widely in current CMB polarization experiments [11], and takes superconducting quantum interference devices (SQUIDs) as the cryogenic readout. The sensors and their readout will be packaged into highly integrated modules, and each module contains 1704 TES sensors. The AliCPT-1 telescope will include four modules, so the number of detectors will reach 6816.

In the second stage, we will have a more sensitive telescope (AliCPT-2) with 12 modules and more than 20 000 detectors. The construction of AliCPT-2 will start in 2020. Each year, we will install four modules and finish the 12 modules by the end of 2022. In Table 1, we summarize the basic instrumental parameters used for the simulations and the corresponding schedule of AliCPT-1 and AliCPT-2.

**Table 1.** Instrumental parameters for AliCPT-1 and AliCPT-2. Schedule for the number of modules (*N*_mod_) and detectors (*N*_det_) installed. NET means the noise-equivalent temperature of each detector; *f*_sky_ represents sky coverage.

Year	2019(AliCPT-1)	2020(AliCPT-1+AliCPT-2)	2021(AliCPT-1 + AliCPT-2)	2022(AliCPT-1 + AliCPT-2)
$\mathrm{NET}\,(\mu \mathrm{K} \sqrt{s})$	350	350	350	350
$N_{\mathrm{\rm {mod}}}$	4	4+4	4+8	4+12
*N*_det_	6816	13 632	20 448	27 264
*f*_sky_	10%	10%	10%	10%
Bands (GHz)	95 $\&$ 150	95 $\&$ 150	95 $\&$ 150	95 $\&$ 150

The scientific goals include:

1. Start large coverage surveys in the northern hemisphere and search for regions with low foreground contamination;
2. Target the cleanest sky region and detect PGWs in the northern hemisphere;
3. Measure the CMB rotation angle with high precision and test the CPT symmetry;
4. Study the hemispherical asymmetry in combination with experiments in the southern hemisphere;
5. Measure E-mode polarization with high precision and study the effects in cosmology;
6. Study cross-correlation of CMB polarization with large-scale structures (LSS).

In the following, we will describe the science covered by AliCPT and provide our preliminary simulations on two main aspects of measurements on *r* and the CMB rotation angle.

### Sensitivity of *r* and its implications for early-Universe physics

The leading scenario for the early Universe is inflationary cosmology, which describes an accelerating expanding phase occurred before the radiation epoch. Inflation resolves several conceptual issues of the Big Bang theory, including the flatness, monopole and horizon problems [12]. Moreover, inflation explains the origin of primordial perturbations, with a mechanism that the quantum fluctuations of the inflaton field were stretched to be classical perturbations by exponential expansion of space. The primordial perturbations have three types: scalar, vector and tensor. Scalar modes will eventually seed the CMB temperature anisotropies and also lead to the formation of large-scale structures in the Universe. Tensor modes, which are dubbed PGWs, will introduce CMB B-mode polarization, which is the target signal in AliCPT observations. Conventionally, we often use *r* to describe tensor perturbations, where *r* means the ratio of the amplitudes of the power spectra of the primordial tensor *A*_T_ and scalar modes *A*_S_, via

(1) \begin{eqnarray*} r \equiv \frac{A_\mathrm{T}}{A_\mathrm{S}}. \end{eqnarray*}

We have performed simulations to forecast the constraining capability of the AliCPT survey. To obtain a constraint on *r* derived from the AliCPT observations, we adopt the Fisher matrix approach [13], which is an efficient way to forecast constraints on cosmological parameters given the specification of the instruments.

Generally, the likelihood function of a multivariate Gaussian-distributed data vector $\mathbf {d}$ can be expressed as,

(2) \begin{equation*} \mathcal {L} = \frac{1}{\sqrt{|\mathbf {C(\theta )}|}} \exp \left(-\frac{1}{2}\mathbf {d^{\dagger }[\mathbf {C}(\theta )]^{-1}}\mathbf {d}\right), \end{equation*}

where θ is the parameter vector and $\mathbf {C}$ is the covariance matrix, which is a function of θ in general. The Fisher matrix, *F*_*ij*_, which is the second partial derivative of the likelihood function with respect to parameters θ_*i*_ and θ_*j*_ evaluated in the fiducial model, approximates the Hessian matrix, and the inverse of the diagonal terms (**F**^−1^)_*ii*_ provides an estimate of the lower limit of the variance for parameter θ_*i*_, i.e. $\Delta \theta _i\gtrsim ({\bf F}^{-1})_{ii}^{1/2}$ [13].

For the case of CMB, $\mathbf {d} = \lbrace X^{\nu }_{\ell m}, ...\rbrace$, where X ∈ {*T*, *E*, *B*}, and ν runs over all available frequency bands. Each $X^{\nu }_{\ell m}$ consists of three components, namely, the lensed CMB, the foreground emission and the instrumental noise. The Fisher matrix for the CMB observables is

(3) \begin{equation*} F_{ij} = \sum _{\ell }\frac{2\ell +1}{2}f_{\mathrm{sky}}\mathrm{Tr}\left[\mathbf {C}^{-1}_{\ell }\frac{\partial \mathbf {C}_{\ell }}{\partial \theta _{i}}\mathbf {C}^{-1}_{\ell }\frac{\partial \mathbf {C}_{\ell }}{\partial \theta _{j}}\right], \end{equation*}

where ℓ denotes the order of the multipole, *f*_sky_ is the sky coverage, and $\mathbf {C}_{\ell }$ is the covariance matrix in harmonic space, which can be expressed as

(4) \begin{equation*} \mathbf {C}_{\ell }(X^{\mu }_{\ell m}, Y^{\nu }_{\ell m}) = C^{XY, \mu \nu }_{\ell } + F^{XY, \mu \nu }_{\ell } + N^{XY, \mu \nu }_{\ell }, \end{equation*}

where *C*_ℓ_, *F*_ℓ_, *N*_ℓ_ correspond to spectra of the lensed CMB, foreground and instrumental noise, respectively. We have assumed that the observed temperature and polarization are statistically isotropic, so that terms of different ℓ are independent.

**Figure 5.**
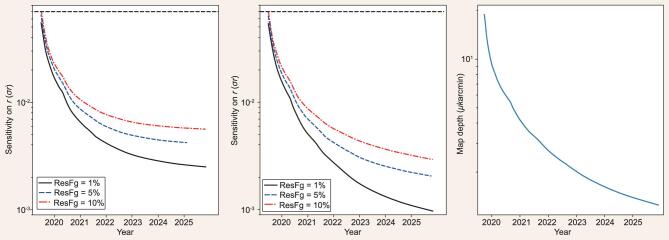
Left panel: Sensitivity of *r*  without delensing (ResFg: residual foreground). Middle panel: Sensitivity of *r*  with complete delensing. The horizontal black dashed line is the current limit *r* < 0.07. Right panel: Map depth of AliCPT.

We use CAMB [14] to calculate the CMB spectra *C*_ℓ_, and choose a fiducial cosmology that is consistent with the Planck 2015 results [15]. Theoretically, the B-mode signal from lensing will exceed that from the primordial signal when, on recombination bump (ℓ ∼ 100), *r* falls below 0.01. Therefore, to achieve high-precision detection of the primordial B-mode signal, we shall perform a delensing procedure to remove the lensing effect from the data. To be clearly, we consider two limit cases, totally delensed and undelensed, in Fig. 5 for comparison. During the actual operation, we will use the data of AliCPT itself, and its cross-correlation with the data of large-scale surveys to reconstruct the lensing effect. We will also attempt to explore the possibility of building a large-aperture CMB telescope at Ali, which will be helpful in delensing.

Based on results of recent experiments, the foreground emission *F*_ℓ_ is thought to dominate over all frequency bands and all scales. This kind of contamination can be removed through multi-frequency surveys, since the frequency spectrum distributions of CMB and other components are different. Component separation is then defined as a method estimating the amount of each emission component. Even when such a separation has been applied, some extent of residual foreground still remains in the data. The AliCPT telescope plans to scan the cleanest sky regions at 95/150 GHz, in order to suppress the residual foreground as much as possible. In our Fisher forecasting, we consider two kinds of components, namely the synchrotron and thermal dust, which contribute to the dominant part of the polarized foreground emission. Besides *r*, we include six additional parameters in the free parameter space, *A*_dust_ and *A*_sync_ for the amplitudes of dust and synchrotron at ℓ = 80 with pivot frequencies of 353 GHz and 23 GHz, α_dust_ and α_sync_ for the spectral index in harmonic space, and β_dust_ and β_sync_ for the spectral index in frequency space, respectively. Also, no conversion between these two components, dust and synchrotron, is assumed in our study.

In our simulations, we assume that the AliCPT instrumental noise of temperature and polarization are uncorrelated, and use an isotropic Gaussian random noise for simplicity. The noise spectrum is given by $N_{\ell }=w^{-1}B_{\ell }^2$ where $B_{\ell } = \exp (-\ell (\ell +1)\theta _{\mathrm{FWHM}}^2/8\log 2)$ is the harmonic transform of the Gaussian beam. The weight is then

(5) \begin{equation*} w^{-1} = \frac{4\pi f_{\mathrm{sky}}\mathrm{NET}^2}{t_{\mathrm{obs}}N_{\mathrm{det}}} , \end{equation*}

where NET means the noise-equivalent temperature, which is closely related to the detector performance, instrument design and atmospheric condition, *t*_obs_ is the effective observation time (from October to March, 12 h per day) and *N*_det_ is the number of detectors. Note that, for polarization, NET should be multiplied by a factor of $\sqrt{2}$ since each polarized signal needs two orthogonal linearly polarized detectors. In Table 1, we show the instrumental parameters for our calculation.

We plot the 1σ sensitivities of *r* and map depth in Fig. 5. Results using the undelensed and totally delensed assumptions are shown in the left and middle panels. Cases related to 1%, 5% and 10% of residual foreground (ResFg) are considered in both panels. Dashed black lines represent the current representative constraint on *r*, *r* < 0.07 at 2σ, obtained from the joint analyses of BICEP2/Keck Array and Planck [16]. As one can see from the plots, even in the undelensed case with 10% residual foreground, after 3 years' survey with AliCPT, the sensitivity on *r* will reach σ_*r*_ = 0.007, which is one order of magnitude stronger than the current constraint. By the end of 2025, after 3 years' observation with 16 detector modules (around 27 000 TES detectors), the constraint on *r* in the delensed case will reach a level of 0.003. Not surprisingly, a lower residual foreground leads to a better result on *r*. If we can suppress foreground down to the 1% level, and apply a delensing procedure, we will obtain σ_*r*_ ∼ 0.001. A much stronger limit of *r* can help us with testing a large number of cosmological models of the early Universe. Due to its high precision, the AliCPT project will provide us with a new vision of the early Universe.

**Figure 6.**
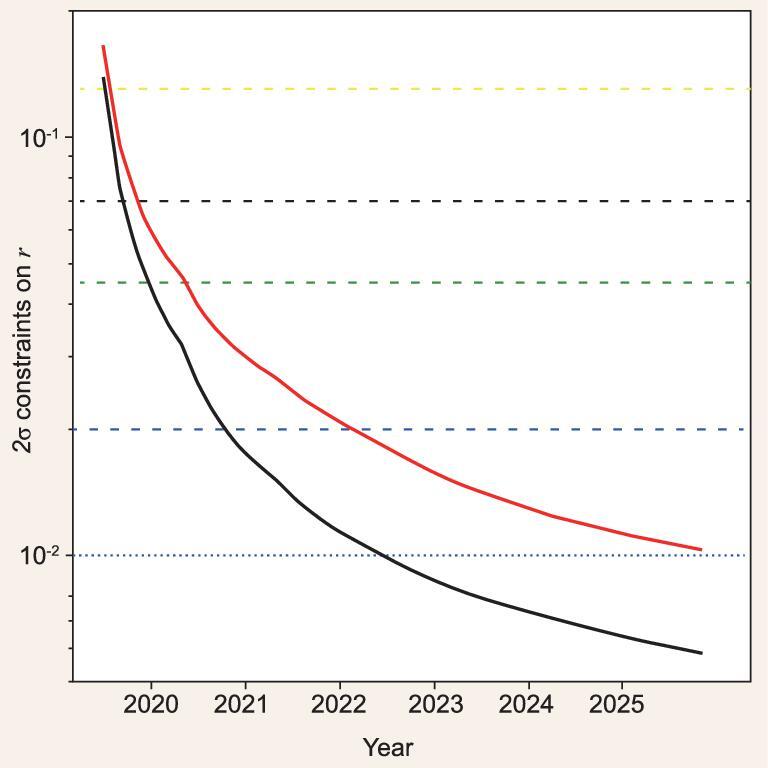
Theoretical predictions of three inflation models and the scheduled AliCPT sensitivity of the measurements on *r*. The black and red curves represent the AliCPT 2σ limits on *r*, where in the simulations we have considered 30% residual lensing effect, and residual foreground of 1% (black) and 10% (red). The black dashed line is the current limit from the BICEP/Keck Array and Planck Collaborations [16]. The yellow and green dashed lines are the predictions from inflation models with potential functions of φ^2^ [17] and φ^2/3^ [18], respectively. The blue dashed and dotted lines are for the alpha-attractor model [19] with α = 7, α = 3 and *n* = 1. In the theoretical calculations, the e-fold number is taken to be 60.

In Fig. 6, we plot the scheduled 2σ constraints on *r* and typical inflation model predictions; we can see that high-precision measurements of PGWs are crucial in testing the inflation models. Since inflationary cosmology still suffers from the initial cosmic singularity [20,21], alternative theoretical approaches have been proposed, including bounce cosmology [22], cyclic Universe [23] and emergent Universe [24]. Similar to inflation, these theories can also generate primordial tensor perturbations. However, PGWs generated from different theories have different characteristics. The precise measurement of the B-mode will help to distinguish between these models of the early Universe.

### Sensitivity of the CMB polarization rotation angle and its implications for the CPT test

Testing CPT symmetry, the combination of charge conjugation (C), parity reflection (P) and time reversal (T) is important to cosmology and particle physics. Any violation, if found, would be a powerful and important clue for new physics beyond the standard model. So far, the laboratory experiments are consistent with a null result for the CPT violation. However, these tests may not be applied to physical processes in the early Universe at extremely high energy scales. In fact, there are motivations to speculate on the CPT violation in cosmology. Firstly, the expanding Universe has a preferred temporal direction, which provides a natural frame to break the Lorentz and CPT symmetries. Secondly, the observed baryon and anti-baryon asymmetry in the Universe may indicate a dynamical CPT violation [25,26].

To study the cosmological CPT violation in CMB, we consider the following effective Lagrangian:

(6) \begin{equation*} \mathcal {L}_\mathrm{CS}=p_{\mu }A_{\nu }\widetilde{F}^{\mu {\nu}}, \end{equation*}

where the external field *p*_μ_ is a constant vector [27], or *p*_μ_ ∼ ∂_μ_φ with φ being the dark-energy scalar in quintessential baryo/leptogenesis [26,28], or *p*_μ_ ∼ ∂_μ_*R* with *R* the Ricci scalar in gravitational baryo/leptogenesis [29,30]; $\widetilde{F}^{\mu \nu }=(1/2)\epsilon ^{\mu \nu \rho \sigma }F_{\rho \sigma }$ is the dual tensor of the electromagnetic tensor *F*_μν_. With the Chern–Simons term in (6), the polarization directions of photons get rotated for CMB; this will convert part of the E-mode polarization to the B-mode and change the power spectra of the polarization fields [31–33]. In Table 2, we summarize the constraints on the rotation angle from various experiments. The current limit on the rotation angle α is about 1°.

**Table 2.** Measurements on CMB rotation angle since 2006.

	Data	$\alpha +\sigma _\alpha ^\mathrm{stat}+\sigma _\alpha ^\mathrm{sys}$
1	WMAP3+BOOMERANG [33]	−6° ± 4°
2	WMAP3 [34]	−2.5° ± 3.0°
3	WMAP5 [35]	−1.7° ± 2.1°
4	WMAP7 [36]	−1.1° ± 1.4° ± 1.5°
5	WMAP9 [37]	−0.36° ± 1.24° ± 1.5°
6	QUaD [38]	−0.56° ± 0.82° ± 0.5°
7	BICEP1 [39]	−2.6° ± 1.02°
8	BICEP1 [40]	−2.77° ± 0.86° ± 1.3°
9	POLARBEAR [41]	−1.08° ± 0.20° ± 0.5°
10	ACTPol [42]	−0.2° ± 0.5°
11	Planck 2015 [43]	0.35° ± 0.05° ± 0.28°

We have performed simulations to forecast the sensitivity of the measurements on the rotation angle with the AliCPTs. For the forecast, we employ the so-called *D*-estimators [38], defined as follows:

(7) \begin{eqnarray*} D_{\ell }^{\mathrm{TB, obs}} &=& C_{\ell }^{\mathrm{TB, obs}}\cos (2\beta ) - C_{\ell }^{\mathrm{TE, obs}}\sin (2\beta )\,\,\nonumber \\ D_{\ell }^{\mathrm{EB, obs}} &=& C_{\ell }^{\mathrm{EB, obs}}\cos (4\beta ) \nonumber\\ &&-\, \frac{1}{2} \left(C_{\ell }^{\mathrm{EE, obs}}-C_{\ell }^{\mathrm{BB, obs}}\right)\sin (4\beta ),\nonumber\\ \end{eqnarray*}

where β is the unbiased estimator for the cosmic rotation angle α.

**Figure 7.**
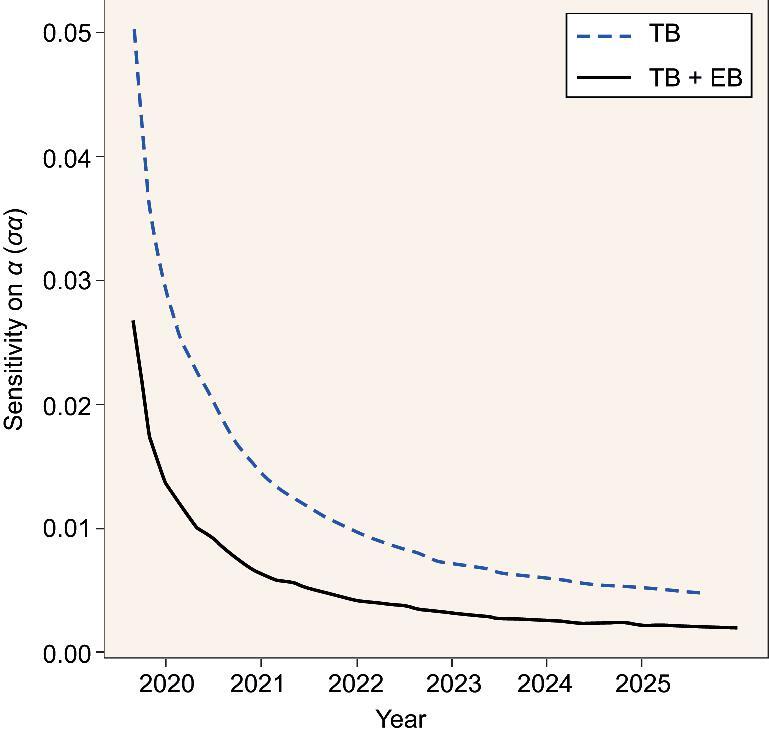
Forecast of the average polarization rotation angle. The blue dashed line is obtained from the TB estimator and the black solid line is from the TB+EB estimator.

We modified the generic Monte Carlo Markov chain (MCMC) sampler provided in the CosmoMC package [44] and performed the calculation with the instrumental properties of AliCPT. We present our results in Fig. 7, where the blue dashed and black solid lines represent the constraints with the TB and EB+TB estimators, respectively. Our results show that, after 3 years' observation, the AliCPTs are capable of providing a stringent constraint on the average rotation angle of σ(α) ∼ 0.01°.

Before concluding, we mention briefly the other topics listed at the beginning of this section. One is the study on the hemispherical asymmetry of CMB polarization, which is also an important scientific goal of AliCPT. After the Planck survey, the temperature anisotropy was precisely measured and well understood, being basically found to be consistent with the standard cosmological model. At the same time, however, some statistical analyses indicate that the CMB temperature suffers from a certain level of asymmetry. For example, this kind of asymmetry can be directly modeled as a dipolar modulation through $\Delta T/T=s(\hat{n})[1+A\hat{n}\cdot \hat{p}]$, where $s(\hat{n})$ refers to a statistically isotropic CMB sky [45]. The best-fit value is *A* = 0.072 ± 0.022 for CMB power at *l* < 64, and pointing to (227°, −27°). There are some explanations for this phenomenon, including systematic errors, galactic contamination and physical origins. If the asymmetry originates from a specific scenario such as the anisotropy of primordial fluctuations, a similar asymmetry pattern should also be observed in CMB polarization. Therefore, measurement of the asymmetry of polarization maps is the key to distinguishing various theories and studying the related physical mechanisms in the early Universe. Combined with the CMB missions in the south, AliCPT will be helpful in realizing a comparison between the polarization maps of different hemispheres, and will play an important role in the investigation of polarized hemispherical asymmetry.

In addition, precise measurement of the CMB E-mode is crucially important to studies on science related to reionization. Due to the large sky coverage, AliCPT will provide us with an excellent opportunity to study the reionization history since the reionization bump corresponds to the CMB spectra with multipoles of ℓ < 10. Also, AliCPT plans to adopt the data of LSS tracers for the cross-correlation studies. The high-precision survey of DESI in the northern hemisphere will be very much aligned with AliCPT for the large overlapping scanning area. Such cross-correlations will provide us with a powerful way to resolve several topics like the reconstruction of the Integrated Sachs-Wolfe (ISW) effect, CMB lensing, etc.

## SUMMARY

In this paper, we have given an introduction to the new ground-based CMB polarization project in China, AliCPT, including the observational conditions and scientific goals. The current AliCPT is located on a 5250 m hilltop in the Ali Prefecture of Tibet, and another site with an altitude of 6000 m is also planned nearby for the forthcoming mission. The median values of PWV crucially important to the CMB experiments at 5250 m and 6000 m are around 1 mm and 0.6 mm from October to March. These are excellent for the observations of CMB polarization at 95/150 GHz (5250 m), and higher frequencies (6000 m). Being in mid-latitudes, AliCPT will cover the whole northern hemisphere and part of the southern hemisphere, and complement the CMB missions in the south, realizing full sky coverage in the search of PGWs.

The major scientific goals of AliCPT include searching for PGWs and measuring the polarization rotation angle. Our preliminary simulations show that, after three observing seasons, AliCPT will improve the current constraint on the tensor-to-scalar ratio by one order of magnitude and the current limit on rotation angle by two orders of magnitude.
